# Characteristics of DOM and Their Relationships with Potentially Toxic Elements in the Inner Mongolia Section of the Yellow River, China

**DOI:** 10.3390/toxics12040250

**Published:** 2024-03-29

**Authors:** Kuo Wang, Juan Jiang, Yuanrong Zhu, Qihao Zhou, Xiaojie Bing, Yidan Tan, Yuyao Wang, Ruiqing Zhang

**Affiliations:** 1 State Key Laboratory of Environment Criteria and Risk Assessment, Chinese Research Academy of Environmental Sciences, Beijing 100012, China; wangkuo21@mails.ucas.ac.cn (K.W.); jiangjuan20@mails.ucas.ac.cn (J.J.); zhouqihao23@mails.ucas.ac.cn (Q.Z.); bingxiaojie0830@foxmail.com (X.B.); tanyidan22@mails.ucas.ac.cn (Y.T.); 2022223082@nwnu.edu.cn (Y.W.); 2 College of Environment, Hohai University, Nanjing 210098, China; 3 School of Energy and Environmental Engineering, University of Science and Technology Beijing, Beijing 100083, China; 4 School of Ecology and Environment, Inner Mongolia University, Hohhot 010021, China; zruiqing2007@126.com

**Keywords:** dissolved organic matter, fluorescence characteristics, FT-ICR MS, heavy metal, interaction, source

## Abstract

The characterization of dissolved organic matter (DOM) is important for better understanding of the migration and transformation mechanisms of DOM in water bodies and its interaction with other contaminants. In this work, fluorescence characteristics and molecular compositions of the DOM samples collected from the mainstream, tributary, and sewage outfall of the Inner Mongolia section of the Yellow River (IMYR) were determined by using fluorescence spectroscopy and Fourier transform ion cyclotron resonance mass spectrometry (FT-ICR MS). In addition, concentrations of potentially toxic elements (PTEs) in the relevant surface water and their potential relationships with DOM were investigated. The results showed that the abundance of tyrosine-like components increased significantly in downstream waters impacted by outfall effluents and was negatively correlated with the humification index (HIX). Compared to the mainstream, outfall and tributaries have a high number of molecular formulas and a higher proportion of CHOS molecular formulas. In particular, the O_5_S class has a relative intensity of 41.6% and the O_5-7_S class has more than 70%. Thirty-eight PTEs were measured in the surface water samples, and 12 found above their detective levels at all sampling sites. Protein-like components are positively correlated with Cu, which is likely indicating the source of Cu in the aquatic environment of the IMYR. Our results demonstrated that urban wastewater discharges significantly alter characteristics and compositions of DOM in the mainstream of IMYR with strongly anthropogenic features. These results and conclusions are important for understanding the role and sources of DOM in the Yellow River aquatic environment.

## 1. Introduction

Dissolved organic matter (DOM—a list of all abbreviations could be found at the end) is widely distributed in aquatic environments such as wastewater, rivers, and lakes [1]. The dissolved organic carbon in DOM accounts for approximately 50% of the total carbon in inland waters and is one of the largest carbon pools [2,3]. DOM is a complex organic mixture containing phenolic, aliphatic, aromatic, and polysaccharide groups, and its geochemical cycling significantly affects the aquatic environment, which also has important implications [4,5]. Microorganisms utilize carbohydrates and amino acids more readily, while humic and fulvic acids are relatively more difficult to be degraded [6,7]. At the same time, DOM also interacts with some nutrients, potentially toxic elements (PTEs), and other contaminants, affecting the transport and transformation processes, bioavailability, and toxicity of these contaminants [8,9,10,11]. Therefore, the effect of DOM on the transport and transformation of PTEs, especially heavy metals in water bodies has received much attention. The binding behavior between DOM and metals binding behavior mainly depends on the nature and type of PTEs [10,12,13].

The sources of DOM has strong heterogeneity, due to the DOM could be generated from the natural environment, such as plant and animal residues [14], and the DOM could be also generated from human activities, such as domestic and industrial wastewater [15,16,17]. Therefore, the molecular compositions, concentrations, and transformations of these DOMs have shown significant differences in spatial and temporal variations in river ecosystems, and which spectral features are effective indicators to identify and characterize the composition and environmental behavior of DOMs [18,19,20]. Among them, the most used methods for DOM compositional characterization include ultraviolet absorption, three-dimensional excitation-emission matrix (3D-EEM), and parallel factor (PARAFAC) analysis, etc. 3D-EEM PARAFAC analysis has been widely used to study DOM and other pollutants in the aquatic environment, including the migration and transformation of organic matter, degradation, and source tracing [21,22,23]. At present, some advanced analytical methods, such as FT-ICR-MS, are also increasingly and widely used in the characterization of DOM in the aquatic environment [24,25,26], which can reveal the compositional characteristics of the whole DOM components and the differences of DOM from various sources at the molecular level compared with 3D-EEM PARAAFA analysis [27,28,29]. However, the convenience and cost of FT-ICR MS detection are limited. Therefore, their joint application is expected to provide a more comprehensive and in-depth analysis of the sources of some pollutants and mechanisms of DOM-metals interactions in watersheds.

The Yellow River is one of the longest rivers in the world and the second-largest river in China. The Yellow River Basin is known for its high sand transport and suspended sediment concentration. It discharges 14.7 billion m^3^ of runoff and 245 million tons of marine sediment annually (1987–2020), accounting for 6% of the total sediment flux from rivers to the ocean worldwide [30,31], and is considered one of the representative watershed systems. Many studies have been conducted on heavy metals and organic pollutants from the Yellow River Basin [32,33,34,35]. Compared with the economically developed watersheds in southern China (i.e., the Yangtze River Basin and the Pearl River Basin), there are fewer systematic studies and reports on the compositional characteristics of DOM in the aquatic environment from the Yellow River. Especially based on the analysis of the compositional characteristics of DOM, few studies have further analyzed the relationships between DOM and the dissolved heavy metals in the water body. The Inner Mongolia section of the Yellow River (IMYR) is located in the middle and upper reaches of the Yellow River basin, where coal mining, coal chemical industry, metal smelting, etc., are the main industrial industries in the region [36,37], At the same time, the Hetao Plain is a crucial irrigated area for grain production and a significant source of agricultural surface pollution [38,39]. The annual wastewater discharge of the Yellow River Basin is 3.376 billion m^3^, and only 48.6% of the Yellow River Basin’s main channels and major tributaries meet water quality standards [40]. The degradation of the basin affects various ecosystem services, and the fragile ecological environment severely restricts the sustainable development of the regional socio-economy.

Therefore, in this study, samples were collected from typical agricultural surface water, sewage outfall, and other water bodies in the IMYR, assuming that there are specific differences in the composition of DOM in these water bodies, which may also have a significant impact on the migration and transformation of PTEs in the Yellow River and may help to analyze and evaluate the sources of pollutants to a certain extent and provide a scientific basis for the management of the water environment. Therefore, the objectives of this study are (1) to characterize the DOM and PTEs compositions of different typical water bodies in the continuous water environment of the IMYR; (2) to analyze the differences in the compositions and distribution characteristics of DOM and PTEs and their interrelationships of the different typical waters; and (3) to explore the possible influence of DOM on the migration and transformation of PTEs in the major rivers as well as the feasibility of tracing the source.

## 2. Materials and Methods

### 2.1. Study Area and Sampling Sites

The IMYR is located in the middle and upper reaches of the Yellow River Basin, entering from Shizuishan City in Ningxia Province and exiting from Jungar Banner, Inner Mongolia, China, with a length of about 843.5 km, accounting for about one-sixth of the total length of the Yellow River. The temperate continental monsoon climate dominates the IMYR, and the region is characterized by low precipitation and high evaporation. The average multi-year temperature is about 9 °C, and rainfall ranges from 150–450 mm/a, mainly concentrated in July to September every year. Due to its unique geographical location, the IMYR is a vital drainage channel for water receding from the local irrigation area and a source of industrial and domestic water for neighboring cities. It plays a vital role in the life and production of the region.

**Figure 1:**
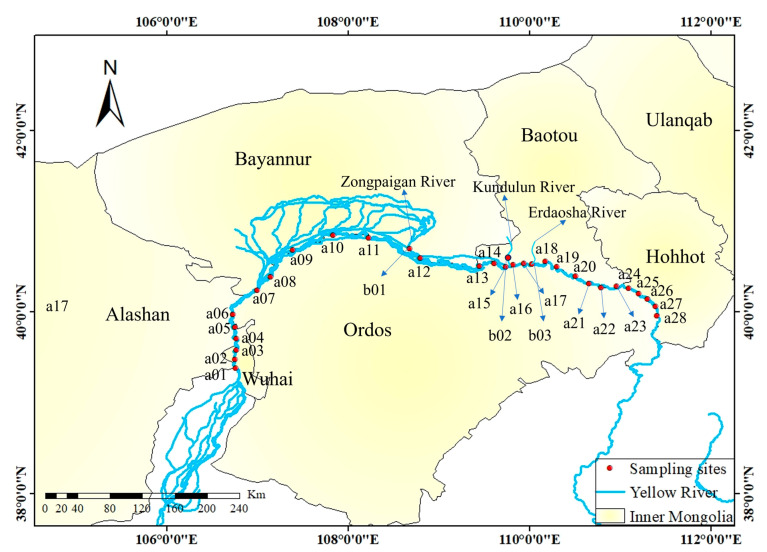
Study area and sampling sites in the Inner Mongolia section of the Yellow River Basin.

Water sampling in the IMYR was conducted in July 2022, and the location and number of sampling points are shown in Figure 1. A total of 31 surface water samples were collected, including 28 points (a01–a28) in the mainstream of the IMYR (from Wuhai City to Hohhot City, China) and three typical sites (b01–b03) in the tributaries. The coordinate information of the sampling sites is given in the Supporting Information (Table S1). Site b01 is the cross-section of the Zongpaigan River, site b02 is the cross-section of the Kundulun River, and site b03 is the outlet of the Tailrace Project and sewage treatment plant of Baotou City located in the Erdaosha River. The Kundulun River was in a flow interruption during the sampling period. Thus, it was not included in the subsequent analysis. Water from the Yellow River main channel through the Sanshenggong Water Conservancy Center Project into the Hetao Plain Irrigation, after irrigation, flows through Wuliangsu Lake and, following the Zongpaigan River, rejoins the Yellow River. As a part of the Baotou City water system, Erdaosha River is currently the centralized discharge channel of Baotou City’s domestic and industrial wastewater, such as rare earth metal smelting and other tailrace projects, which is mainly involved in the production and domestic wastewater of Wanshuiquan Wastewater Treatment Plant and Baotou Rare Earth Hi-Tech Zone. Therefore, in this study, sites b01 and b03 are proposed to be taken as the dividing points of the upstream, midstream, and downstream of the IMYR, i.e., the upstream (a01–a11, *n* = 11), midstream (a12–a17, *n* = 6) and downstream (a18–a28, *n* = 11) of the IMYR, respectively.

Before sampling, polyethylene plastic bottles and samplers were well moistened with river water, and samples were collected below the water surface 0.2 m at each site and stored in sealed containers. Samples were refrigerated and transported to the laboratory as quickly as possible. Water samples were filtered through a 0.45 μm membrane filter, refrigerated, and analyzed for DOM characterization and concentrations of PTEs.

### 2.2. DOM Fluorescence

FDOM in DOM of filtered water samples was determined using an F-7000 fluorescence spectrometer (Hitachi High-Technologies, Tokyo, Japan) with a 1-cm quartz colorimetric tube and Milli-Q water as a blank. The scanning parameters of the instrument were set as follows: the excitation power of the xenon lamp light source was 450 W, the PMT voltage was 700 V, and the scanning speed was 1200 nm/min; the excitation wavelength (Ex) was set to 200–450 nm, the emission wavelength (Em) was set to 250–550 nm, and the slit widths of both the excitation and emission wavelengths were 5 nm.

All raw EEM processing was performed using the drEEM toolbox [21] in MATLAB software 23.2 (The MathWorks Inc., Carlsbad, CA, USA). The EEM calibration process included blank EEM subtraction and scatter removal, correction for internal filter effects [41], and normalization of Raman units (RUs) before PARAFAC analysis. Raman and Rayleigh scattering peaks were removed from the spectra within the drEEM toolbox, as well as wavelengths below λ_ex_ = 250 nm and λ_em_ = 300 nm to limit the spectral noise region of the matrix. Raman normalization factors were calculated at λ_ex_ = 350 nm using a Milli-Q blank EEM [42]. The resulting corrected and normalized EEM data set was subjected to PARAFAC analysis using the drEEM toolbox. Least squares models with three to six components were tested by running 30 iterations with non-negativity and a convergence criterion of 1 × 10^−8^ [22], and the most appropriate number of PARAFAC components was found using a combination of split halves and residuals. The resulting fluorescent components were uploaded to the OpenFluor database [43] (https://openfluor.lablicate.com/, accessed on 30 May 2023) for comparison, limiting the identity coefficient to 0.95, searching for spectral data similar to the present study and plotted (Figure S1).

In addition, several fluorescence indices were calculated. The fluorescence index (FI), commonly used to indicate DOM sources [44] (1.8: microbial sources, 1.2: terrestrial sources [45]), was calculated by the ratio of the fluorescence intensity at Em = 470 nm to that at Em = 520 nm when Ex =370 nm [46]. The humification index (HIX), which is used to assess the degree of DOM humification [47], was defined as the fluorescence intensity in the region from 300 to 345 nm divided by the sum of the intensity in the areas from 300 to 345 nm and from 435 to 480 nm. The biological index (BIX), used to assess the relative contribution of endogenous substances to DOM [23], was calculated by dividing the fluorescence intensity at Em = 380 nm by that at Em = 430 when Ex = 310 nm [48].

### 2.3. FT-ICR MS

To characterize DOM composition at the molecular level, six typical samples were selected for FT-ICR MS analysis, including four mainstream sites (a04, a14, a17, a23) and two major tributary sites (b01, b03). Before analysis, an estimated volume of acidified (pH = 2) sample was prepared to achieve a target concentration of 45 μg-C/mL [49], and solid-phase extraction (SPE) of DOM was performed using PPL Bond Elut resin (Agilent) [50]. SPE-DOM was eluted with 2 mL methanol and dried under nitrogen. The methanol extract was redissolved in 1 mL and analyzed using a 9.4 T SolariX XR FT-ICR MS (Bruker, Berlin, Germany).

The decomposition analysis converted the mass spectral peaks to the corresponding molecular formulas. The modified aromaticity index (AI_mod_) [29], double bond equivalents (DBE), the island of stability (IOS) [24], the molecular lability boundary for more labile contributions (MLB_L_) [25], H/C and O/C ratios were calculated. The molecular components in the mass spectrometry data were classified into different types of compounds according to the previous classification method, which was limited by different parameter ranges [51,52,53]: polycyclic aromatics (PA, AI_mod_ > 0.66); highly aromatic compounds (HA, 0.5 < AI_mod_ ≤ 0.66); highly unsaturated compounds (HU, AI_mod_ ≤ 0.5, H/C < 1.5); unsaturated aliphatic compounds (UA, 1.5 ≤ H/C ≤ 2, N = 0); peptides (1.5 ≤ H/C ≤ 2, N > 0) and carboxyl-rich alicyclic molecules (CRAM; 0.3 < DBE/C < 0.68, 0.2 < DBE/H < 0.95, 0.77 < DBE/O < 1.75) [54].

### 2.4. Potentially Toxic Elements

Water samples obtained after filtration through a 0.45 μm membrane were analyzed by inductively coupled plasma mass spectrometry (7800 ICP-MS, Agilent, Santa Clara, CA, USA) for all-element metal screening and systematically analyzed for validly detected PTEs above the detection limit.

The detection limits of the tested elements are shown in Table S2. In order to ensure the quality and accuracy of the elemental detection, Rh, Re and Th were added as internal standards in the experimental process, and the recoveries of the different internal standards were (90.1~120.9%), (98.6~113.5%) and (93.9~107.7%), respectively; in addition, some repeated analytical tests were carried out in the middle of sample testing as well as after the end of sample testing, and the standard deviation of each element in two repeated analytical tests was calculated. In addition, some samples were repeatedly analyzed in the middle and at the end of the sample testing, and the standard deviation of each element in the two repeated analytical tests was calculated, which is shown in Table S2.

### 2.5. Statistical Analyses

Sampling site locations were plotted using ArcGIS 10.8. Statistical analyses were performed using IBM SPSS Statistics 27 (IBM Corp., Armonk, NY, USA), and plots were generated using Origin 2022 with MATLAB and other software. Pearson correlation analysis was used to identify PTEs with characteristics closely related to DOM. Linear regression analysis examined the linear relationships between fluorescence indices and fluorescence components. Principal component analysis (PCA) was used to analyze the fluorescence components, fluorescence indices, and PTEs. Additionally, PTEs below the detection limit and those with poor correlation were excluded from the PCA analysis.

## 3. Results and Discussion

### 3.1. Fluorescence Characteristics of DOM

**Figure 2:**
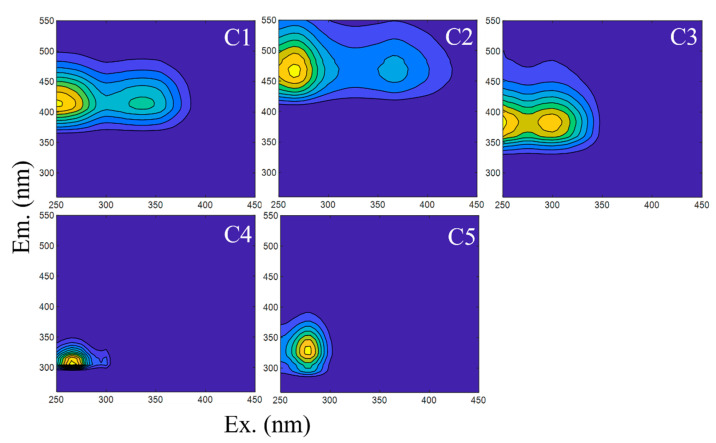
Fluorescent components of DOM in the water body from the Inner Mongolia section of the Yellow River Basin.

Five fluorophore components were identified using PARAFAC analysis (Figure 2). These include three humic-like components (C1–C3) and two protein-like components (C4–C5). According to the fluorophore species classification by Coble [55,56], C1 [Ex/Em = 250(335)/415] and C2 [Ex/Em = 265(365)/465] consist of a combination of UVC humic-like peak A and UVA humic-like peak C. These components predominantly feature peak A, associated with abundant natural humic acids and fulvic acid fluorophores [57,58]. The component C3 [Ex/Em = 250(295)/380], corresponding to peak M, is identified as a microbial humic-like component [57,59]. C4 [Ex/Em = 265/305] corresponds to the tyrosine-like component related to peak B [58]. Additionally, C5 [Ex/Em = 275/330] is analogous to the tryptophan-like component, corresponding to peak T [55]. Both protein-like components, C4 and C5, are commonly observed in water bodies influenced by wastewater [45,60].

**Figure 3:**
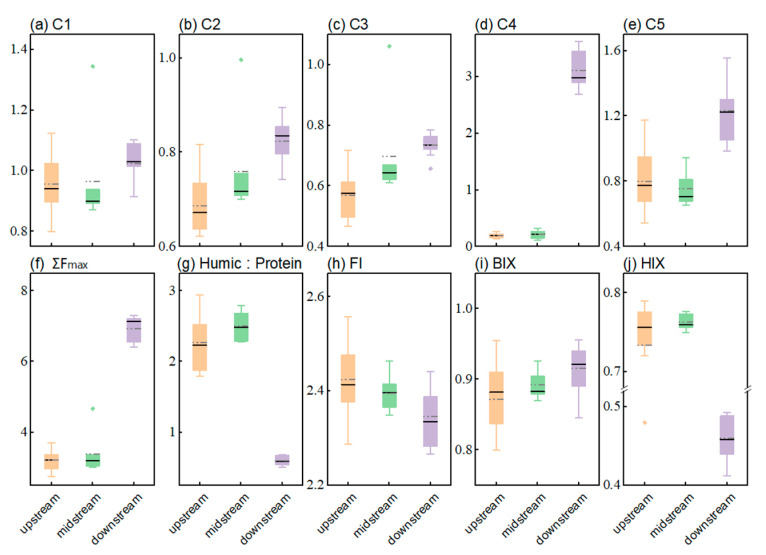
Fluorescent components (C1–C5) (**a**–**e**), sum of fluorescent component contents (C1 + C2 + C3 + C4 + C5) (**f**), The ratio of humic-like components (C1 + C2+ C3) to the protein-like components (C4 + C5) (**g**), the fluorescence intensity (**h**), biological index (**i**) and humification index (**j**) content distribution in the upper, middle and lower reaches of the Inner Mongolia section. The solid black line represents the median, and the dashed gray line represents the mean.

The abundance of each fluorescent component in the IMYR showed a general increasing trend from upstream to downstream, as shown in Figure 3. It is noteworthy that the protein-like components (C4, C5) showed a strong spatial variability, especially the abundance of the tyrosine-like component C4 was much higher in the downstream than in the upstream and midstream (Figure 3d), with a mean value of approached 15 times higher than that observed in the upstream and midstream (Table S3). Differences in fluorescent components and fluorescence indices between the upstream and midstream of the IMYR were not pronounced, with the value changes of each index generally falling within the same range (Figure 3). This suggests that the water from Zongpaigan River, after being used for irrigation and discharged into the mainstream via Wuliangsu Lake, probably had minimal impact on the DOM Characteristics of the mainstream in the sampling period. Erdaosha River, where the b03 site is located, is the boundary between the midstream and downstream of IMYR divided in this study. It is also a tributary of the Yellow River but receives sewage effluents from the wastewater treatment plant in Baotou City as described in Section 2.1. The b03 site was excluded as an outlier in the PARAFAC analysis due to its higher fluorescence peak intensity (Ʃpeak of 12.89) compared to the upstream (4.80 ± 0.44), midstream (5.06 ± 0.79), and downstream (7.98 ± 0.43) (Table S3). Studies using the excitation-emission matrix (EEM) have identified distinct fluorescence characteristics in sewage effluents compared to unaffected rivers [61,62]. Tryptophan-like and tyrosine-like components (C4 and C5 in this study) are commonly used to indicate anthropogenic influences such as wastewater treatment plants [63,64]. Therefore, it could be concluded that the DOM abundance, especially protein-like components, in the Erdaosha River, where the b03 site is located, has increased significantly due to wastewater inputs. Meanwhile, since the wastewater discharged from the Erdaosha River outfall contains domestic and industrial wastewater, it is difficult to identify the specific source using fluorescence spectroscopy without other analytical tools.

The abundance of protein-like components increased considerably in the downstream waters, leading to higher ΣFmax than the upstream and midstream (Figure 3f). This shift is further elucidated by the ratio of humic-like to protein-like components [26], which revealed that humic-like components predominated upstream and midstream, while protein-like components were more prevalent downstream (Figure 3g). This finding is consistent with the results of the H: P ratio. Notably, the effluents of underutilized wastewater treatment plants often contain unprecipitated bioflocs composed of microorganisms and their extracellular polymers [65,66]. These bioflocs may, under certain conditions, undergo cell hydrolysis accompanied by lysis of cell [67], producing soluble microbial products (SMP) [68], which have been identified as biomass-associated products (BAP) [69], leading to an increase in the abundance of protein-like components. Therefore, it can be hypothesized that the increase in abundance of C4 and C5 components may be related to incompletely decomposed components from wastewater [70]. A comprehensive analysis suggests that the substantial increase in the abundance of protein-like components in downstream waters is likely linked to the discharge of Baotou’s urban domestic and industrial wastewater, which is likely inadequately treated. The results indicate that urban production and domestic wastewater discharge are significant point sources of protein-like components in the water from IMYR.

**Figure 4:**
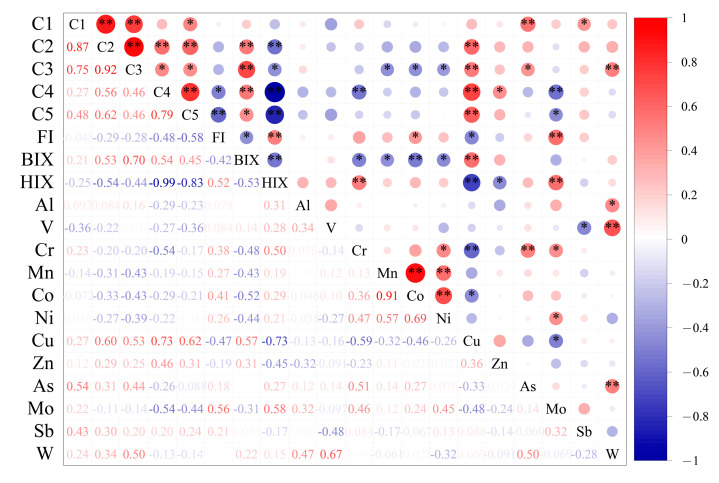
Correlation analysis between potentially toxic elements and DOM fluorescence components and related indexes in water in the Inner Mongolia section (** represents *p* value < 0.01, * represents *p* Value < 0.05; the number in the figure represents the correlation coefficient r).

**Figure 5:**
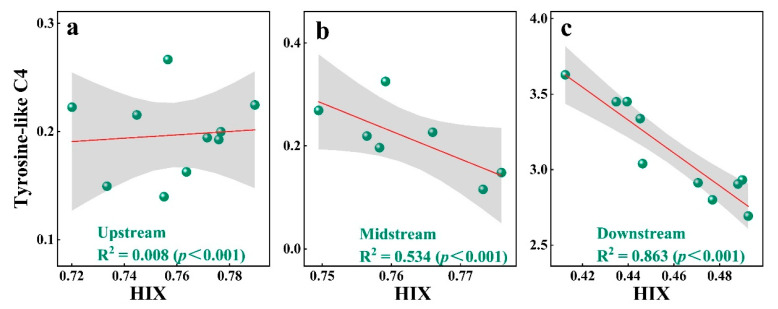
Linear relationships between HIX and Tyrosine-like components C4 in the upstream (**a**), midstream (**b**), and downstream (**c**) of the IMYR.

For all water samples from IMYR, FI values ranged from 2.2 to 2.9, suggesting a significant microbial contribution to DOM in the IMYR (Figure 3h). Duan et al. [71] pointed out a positive correlation between fluorescence index (FI) and point source pollution, and which concluded that FI can be used to realize the qualitative analysis of pollution sources. However, the opposite situation was observed in this study, FI was negatively correlated with protein-like components (C4, C5) representing point source pollution (Figure 4). The HIX values at all sites were below 0.8, indicating the humification of DOM was low in water from IMYR, Especially, HIX values in the downstream (0.46 ± 0.03) was lower than that in the upstream (0.76 ± 0.02) and midstream (0.76 ± 0.01) (Table S3). The present study found a significant negative correlation between HIX and tyrosine-like components (C4) (Figure 4). In response to this finding, the linear relationship between HIX and tyrosine-like components was further explored (Figure 5). In contrast to the Pearson correlation analysis performed for the whole mainstream from IMYR, the linear regression analysis performed by different divided section of the IMYR in this study showed more clearly that the linear relationship between HIX and tyrosine-like C4 is significant in the downstream (Figure 5c).

Aggregative results showed that HIX and tyrosine-like substances are more strongly associated in the effluent-impacted downstream than that less impacted in the upstream and midstream of the IMYR. Thus, this study indicated that the linear relationship between tyrosine-like substances and the humification index could be used to assess water pollution levels.

### 3.2. Molecular Characteristics of DOM

In the comprehensive review of the results from the molecular analysis of DOM across the mainstream and tributary sampling sites, as shown in Table 1, no obviously differences in molecular characterization parameters were found among the four mainstream sampling sites (a04, a14, a17, a23), which is likely representing general results from the mainstream. However, notable differences were observed between the mainstream and tributary sites. Specifically, the tributary sites exhibited a higher number of molecular formulas than that in the mainstream sites. The b03 site at the Erdaosha River showed distinct characteristics from other sites: The molecular weight (MW) was lower than the average value from mainstream, which potentially due to enhanced microbial degradation of effluent leading to an increase in the low molecular weight of DOM [72]. Additionally, the values of DBE and AI_mod_ were lower than those average values from the mainstream, suggesting that the DOM is less unsaturated and aromatic in the effluent. In addition, higher MLB_L_ and lower IOS indicated that wastewater input to the water contributes to the increased DOM instability of the water in the IMYR.

**Figure 6:**
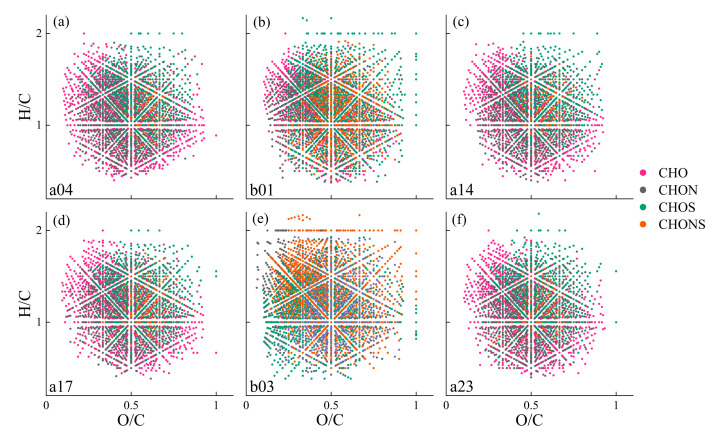
The upstream point a04 (**a**) of the Inner Mongolia section, the dividing point between the upstream and the midstream is b01 (**b**), the midstream point a14 (**c**) and a17 (**d**), the dividing site between the midstream and the downstream b03 (**e**) and the downstream point a23 (**f**) Van Krevelen diagram of the molecular formulas of CHO, CHOS, CHON, and CHONS.

Van Krevelen (V-K) plots, which visualize the FT-ICR MS data, revealed the distributions of four molecular groups, as shown in Figure 6. The proportions of six compounds are illustrated in Figure S2. Within the 200 to 600 Da mass range, the molecular formulas at the four sites from the mainstream predominantly consisted of CHO (46.57% ± 0.28%) and CHON (39.39% ± 0.48%), accounting for over 85% of the total molecular ratio. The relative intensities of CHO in these samples varied from 72.66% to 74.73%. In contrast, the tributary sites (b01 and b03) exhibited significantly higher proportions of CHOS (16.33%, 18.16%) and CHONS (8.78%, 8.05%) compared to the mainstream average (CHOS = 11.49% ± 0.44%, CHONS = 2.56% ± 0.29%). Furthermore, the sites from midstream (a14, a17) appeared unaffected by the tributary where site b01 is located, which is consistent with the results from fluorescent components and abundance analysis. Notably, the relative intensity of CHOS was highest at the b03 site that accounted for 28.09%, which was much higher than that in the mainstream with an average value of 6.66% ± 0.28%. Previous studies have indicated that CHOS molecular formulas could constitute 5% to 20% of the DOM in natural water [73,74]. At site b03, the sulfur-containing molecular formula predominantly belonged to the O_1-13_S class, with the O_5_S class showing a relative intensity of 41.6% and the O_5-7_S class exceeding 70%. Previous research suggested that the O_5_S class in CHOS was related to linear alkylbenzene-sulfonates (LAS) and their degradation products, sulfophenyl carboxylic acids (SPC) [75,76], which are synthetic surfactants commonly found in detergents and personal care products and widely detected in wastewater and also other anthropogenically impacted inland waters [77,78]. These observations indicated that the increased proportion of CHOS in the DOM at the b03 site, particularly in the O_5_S category, was attributable to wastewater discharge from the Erdaosha River outfall. Thus, the aggregative results suggest that human activities significantly influence the variation in CHOS levels between the mainstream and the tributary.

**Figure 7:**
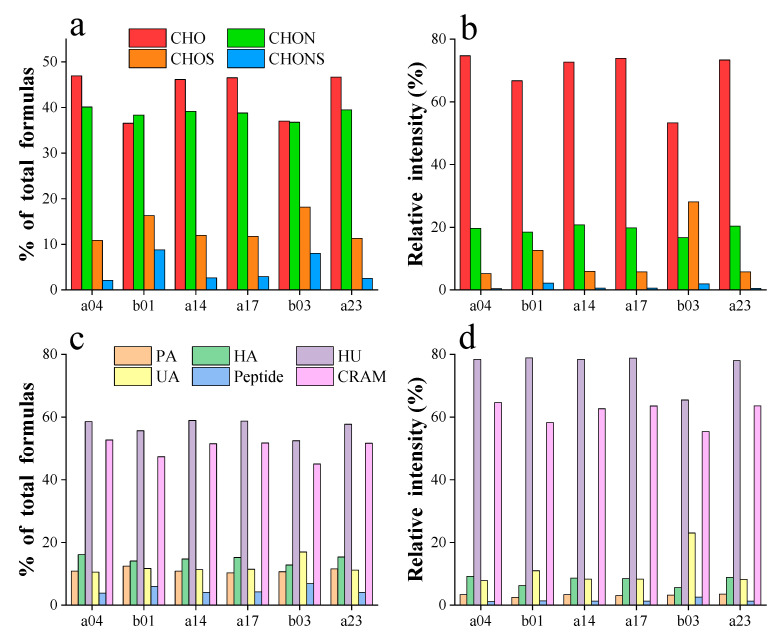
The proportion of the number of different molecular formulas (**a**) and the proportion of relative intensities (**b**), the proportion of the number of different molecular types (**c**) and the proportion of relative intensities (**d**) of 6 DOM samples.

The majority of molecular formulas in this study were classified as highly unsaturated compounds (HU, 57% ± 2.31%) and carboxyl-rich alicyclic molecules (CRAM, 49.99% ± 2.8%), with relative abundances of 76.3% ± 4.87% and 61.33% ± 3.36%, respectively, as shown in Figure 7c,d. The CRAM, widely recognized in the DOM of natural water that used as an indicator of stability of DOM [54,79], which was less abundant at the tributary sites. This suggested lower stability of DOM at tributary sites, consistent with the results from the IOS value. Unsaturated aliphatic compounds, typically derived from microbial metabolites [80] and indicative of biologically unstable DOM sources [81], were particularly abundant in the sample from b03 site. Here, the relative abundance (16.96%) and intensity (23.03%) were much higher than those average values form the mainstream (11.13% ± 0.35%, 8.19% ± 0.19%). These observations indicated that intense microbial activity in wastewater discharged from the outfall would result in elevated levels of unsaturated aliphatic compounds as microbial metabolites, as detailed in Table S4. These results further suggested that the urban domestic and industrial wastewater would affect the compositional characteristics, and thus also influence the transport and transformation patterns of DOM in the IMYR.

### 3.3. Concentrations and Distribution Characteristics of PTEs in Water

Based on the PTEs screening and quantification in the IMYR waters, the total detection rate for 15 rare earth elements was 6.7%. The detection rates for five heavy metals, namely Ti, Ga, Ge, Zr, and Pb, were less than 20%. Pd (detection limit: 0.02 μg/L), Cd (detection limit: 0.05 μg/L), Sn (detection limit: 0.08 μg/L), and Tl (detection limit: 0.02 μg/L) were not detected in any of the samples. Concentrations of twelve PTEs could be detected in the samples from all sites, with average concentrations (μg/L) in descending order that Cr (4.109) > Mn (3.930) > As (3.154) > Mo (2.549) > Al (2.078) > V (1.819) > Zn (1.669) > Cu (1.414) > Ni (1.309) > Sb (0.972) > W (0.191) > Co (0.146), as detailed in Table S5.

**Figure 8:**
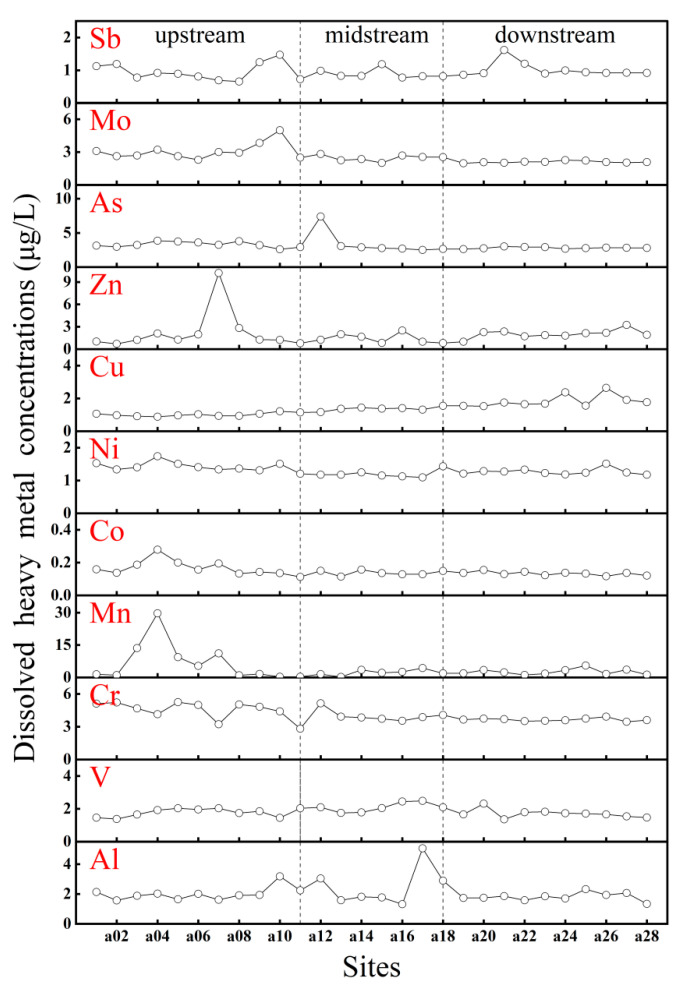
Spatial distribution of PTEs concentrations (μg/L) in water in the Inner Mongolia section. The black dotted line represents the dividing line between the upper, middle, and lower reaches of the Inner Mongolia section.

Spatial variations in PTEs concentrations were observed in the surface water of the IMYR (Figure 8). The average concentration of Cu showed an increasing trend from upstream to downstream. Mo concentrations increased upstream but remained relatively stable in the middle and downstream. Conversely, the average concentration of Cr decreased from upstream to downstream, with fluctuations in the upstream. Notably, sites with higher concentrations of Mn and Co were in the upper reaches of the IMYR, with a similar spatial distribution. A significant positive correlation between Mn and Co (r = 0.91, *p* < 0.01) was identified (Figure 4), suggesting common sources of contamination or similar migration and transformation processes for these two metals in the IMYR [82].

The upper reaches of the IMYR are rich in mineral resources, with numerous open-pit coal mines. Dust from coal mining and transportation entering the Yellow River through atmospheric deposition may contribute to fluctuations in concentrations of PTEs. Heavy industries in the region, especially metal smelting and coal chemical industries, are known for high pollution before [83]. Industrial wastewater discharges could explain the elevated and fluctuating concentrations of Mn, Co, and Cr in the upper reaches. In addition, the extensive agricultural practices in the Hetao Plain involve using fertilizers, pesticides, and herbicides containing PTEs. These substances can accumulate in the soil and migrate to the water of Yellow River via rainfall and irrigation [84]. Other PTEs, such as Ni and V, did not show significant changes in concentration, suggesting that natural background levels and the headwaters of the Yellow River is likely a primary influence factor for their presence.

### 3.4. Implication for Interaction and Source of DOM and PTEs

The correlation matrix reveals significant relationships between various fluorescent components and PTEs (Figure 4). The humic-like component C1 exhibited a significantly positive correlation with the PTEs of As (*p* < 0.05). The microbial humic-like component C3 negatively correlated with the PTEs of Mn, Co, and Ni (*p* < 0.05). A significant positive correlation was also observed between the humification index (HIX), Cr, and Mo. The tyrosine-like component C4 was negatively correlated with the PTEs of Cr and Mo, suggesting a potential fluorescence quenching effect of these metals on C4 or a complex relationship with their sources, warranting further investigation.

Previous studies reported Cu complexation with DOM, leading to fluorescence quenching [85,86,87]. However, a positive correlation between the PTEs of Cu and fluorescent components was shown in this study (Figure 4), which could imply limited Cu-DOM complexation due to competition with other ions. Or else, it might reflect a close relationship between the sources of Cu and protein-like components. The intense microbial activity in the effluent from the Erdaosha River outfall, leading to an increase in protein-like components, did not diminish with rising Cu concentrations, further suggesting a potential link between Cu sources and protein-like components.

**Figure 9:**
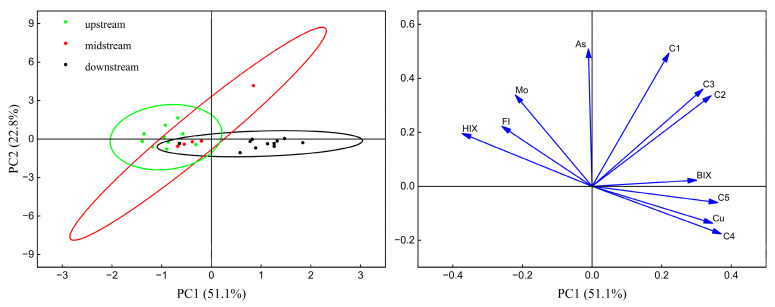
Principal component analysis (PCA) for fluorescence components, related indexes, and potentially toxic elements (Cu, As, and Mo).

Three potentially toxic elements, Cu, As, and Mo, showing significant correlations with fluorescent components, were selected for principal component analysis (PCA) (Figure 9). The cumulative variance explained by the principal components was 73.9%, with the first principal component (PC1) accounting for 51.1% of the total variance. PC1 predominantly comprised eight indicators: fluorescent components C2, C3, C4, C5, fluorescence indices FI, HIX, BIX, and Cu. Compared to upstream sampling sites, the higher PC1 values at downstream sites in the IMYR are consistent with the observed higher abundance of fluorescent components downstream (Figure 3a–e). PC1 also contained Cu that changes in its concentration were consistent with changes in the abundance of each fluorescent component along the mainstream. No decrease in the abundance of the fluorescent components due to Cu-DOM complexation was observed. This may suggest that some fluorescent components (protein-like) could be the indicator for the source of Cu in the aquatic environment of the IMYR. The negative loadings of FI and HIX in PC1 are consistent with previous analyses, suggesting an increase in microbially derived DOM and a decrease in humification due to intensely microbial activity in wastewater from the Erdaosha River outfall.

PC2, contributing 22.8% to the total variance, included the humic-like component C1 and As and Mo. The component C1, characterized as ubiquitous terrestrial humic-like and absent in wastewater DOM, which did not show a significant spatial distribution trend in this study (Figure 3a) and was not notably elevated due to downstream wastewater discharge. This observation is consistent with its absence in wastewater DOM. Although the Zongpaigan River, where the b01 site is located, carries agricultural irrigation water, the study could not conclusively link PC2 to agricultural influences. The spatial distribution trends of As and Mo concentrations mirrored those of the C1 component; the humic-like C1 component may indicate the sources of As and Mo in water form IMYR, further investigation is needed to determine specific sources. In summary, PC1 appears more influenced by anthropogenic factors, while PC2 may represent the Yellow River upstream water sources and natural background influences.

## 4. Conclusions

The Zongpaigan River, which carries water from agricultural irrigation activities in the Hetao Plain, has minimal impact on the characteristics and composition of the DOM in the mainstream during sampling period in this study. However, urban industrial and domestic wastewater, which is a significant point source of pollution in the IMYR, affects the abundance of protein-like components, especially tyrosine-like components. The results of linear regression analysis showed a significant linear correlation between HIX and tyrosine-like components in the water influenced by wastewater discharges, which linear correlation can be used to assess the water pollution level. The FT-ICR MS analysis revealed that the tributary DOM is characterized by higher instability, lower unsaturation, and lower aromaticity. Especially the water from the Erdaosha River, the sulfur molecular formulas in the O_5_S class accounted for 41.6%, and the O_5-7_S class accounted for exceeds 70%. This pattern may be a crucial indicator of concentrated urban industrial and domestic wastewater discharge.

In the water from IMYR, twelve PTEs were detected in all sampling sites. Spatial variations in the concentrations of some PTEs may occur due to dust from open-pit mining, industrial discharges, and agricultural activities. Contrary to the traditional results of the quenching of fluorescence by Cu, there was a significant positive correlation between Cu and protein-like components in the waters of the IMYR. This correlation may be closely related to the influx of protein-like substances in municipal wastewater and sources of copper in regional sewage, such as metal smelting.

This study sheds light on the spatial variation of DOM components in the IMYR, especially in downstream areas which were severely impacted by urban wastewater discharges. The findings reported in this work are crucial for understanding the role and sources of DOM in the Yellow River aquatic environment and helpful for further research on the pollutants, such as PTEs, and their migration and transformation. As the IMYR is characterized by high sediment loading and a substantial amount of suspended particulate matter in the water body, a researchable scientific question is how these factors might influence DOM composition and its interaction with pollutants. Future research should address these issues to improve our understanding of organic matter characteristics in large river systems and their impact on contaminant sources, migration, and transformation.

## Figures and Tables

**Table 1:** Molecular characterization of DOM from FT-ICR MS (Intensity-weighted methods were applied to calculate the average MW, DBE, AI_mod_, and H/C and O/C ratios).

	a04	b01	a14	a17	b03	a23
Number of formulas	4402	6719	4026	4194	6620	4166
Average MW (Da)	365.39	366.17	359.04	359.65	346.92	359.79
Average H/C	1.20	1.25	1.21	1.21	1.33	1.21
Average O/C	0.50	0.52	0.51	0.51	0.45	0.51
Average DBE	7.97	7.47	7.73	7.72	6.61	7.78
Average AI_mod_	0.33	0.28	0.32	0.32	0.26	0.32
MLB_L_ (%)	14.5	17.8	15.5	15.7	24.1	15.3
IOS (%)	7.3	6.8	7.5	7.4	6.1	7.2

## Data Availability

The original contributions presented in the study are included in the article/Supplementary Material, further inquiries can be directed to the corresponding author.

## Supplementary Materials

The following supporting information can be downloaded at: https://www.mdpi.com/article/10.3390/toxics12040250/s1, Table S1: The coordinates of the sampling sites. Table S2: Element detection limit, Two-time element concentration data (Site a18, b03) and their standard deviation. Table S3: Descriptive analysis of DOM fluorescence components and related parameters in the upper, middle, and lower reaches of the Inner Mongolia section. Table S4: The proportion of the number and relative intensity of different molecular formulas and different molecular species in the six DOM samples relative to the total number of molecules and the total intensity. Table S5: Distribution of heavy metal concentrations in the upper, middle, and lower reaches of the Inner Mongolia section of the Yellow River Basin. Figure S1: Comparison of a five component PARAFAC model with other models in the OpenFluor database. Figure S2: The upstream site a04 (a) of the Inner Mongolia section, the dividing point between the upstream and the midstream is b01 (b), the midstream site a14 (c) and a17 (d), the dividing point between the midstream and the downstream b03 (e) and the downstream site a23 (f) Van-Krevelen diagram of DOM molecular species.

## Abbreviations

DOM	dissolved organic matter
IMYR	Inner Mongolia section of the Yellow River
PTEs	potentially toxic elements
PARAFAC	parallel factor analysis
FI	fluorescence index
BIX	biological index
HIX	humification index
AI_mod_	modified aromaticity index
DBE	double bond equivalents
IOS	island of stability
MLB_L_	molecular lability boundary for more labile contributions
FT-ICR MS	Fourier transform ion cyclotron resonance mass spectrometry
