# Supplementary material for: Characteristics of DOM and Their Relationships with Potentially Toxic Elements in the Inner Mongolia Section of the Yellow River, China
Source: Toxics. 2024 Mar 29;12(4):250. doi: 10.3390/toxics12040250 (PMC11054287; doi:10.3390/toxics12040250)
Supplement: Supplementary file 1 [file toxics-12-00250-s001.zip › toxics-2933544-supplementary.pdf]

Supplementary Materials

Characteristics of DOM and Their Relationships with Potentially Toxic Elements in the Inner Mongolia Section of the Yellow River, China

Kuo Wang [1], Juan Jiang [1,2], Yuanrong Zhu [1,*], Qihao Zhou [1], Xiaojie Bing [1,3], Yidan Tan [1], Yuyao Wang [1] and Ruiqing Zhang [4]

[1] State Key Laboratory of Environment Criteria and Risk Assessment, Chinese Research Academy of Environmental Sciences, Beijing 100012, China; wangkuo21@mails.ucas.ac.cn (K.W.); jiangjuan20@mails.ucas.ac.cn (J.J.); zhouqihao23@mails.ucas.ac.cn (Q.Z.); bingxiaojie0830@foxmail.com (X.B.); tanyidan22@mails.ucas.ac.cn (Y.T.); 2022223082@nwnu.edu.cn (Y.W.)
[2] College of Environment, Hohai University, Nanjing 210098, China
[3] School of Energy and Environmental Engineering, University of Science and Technology Beijing, Beijing 100083, China
[4] School of Ecology and Environment, Inner Mongolia University, Hohhot 010021, China; zruiqing2007@126.com
* Correspondence: zhuyuanrong07@mails.ucas.ac.cn

Contents of this file:

1. Supplementary figures: Figures S1 to S2
2. Supplementary tables: Tables S1 to S5

1. Supplementary figures

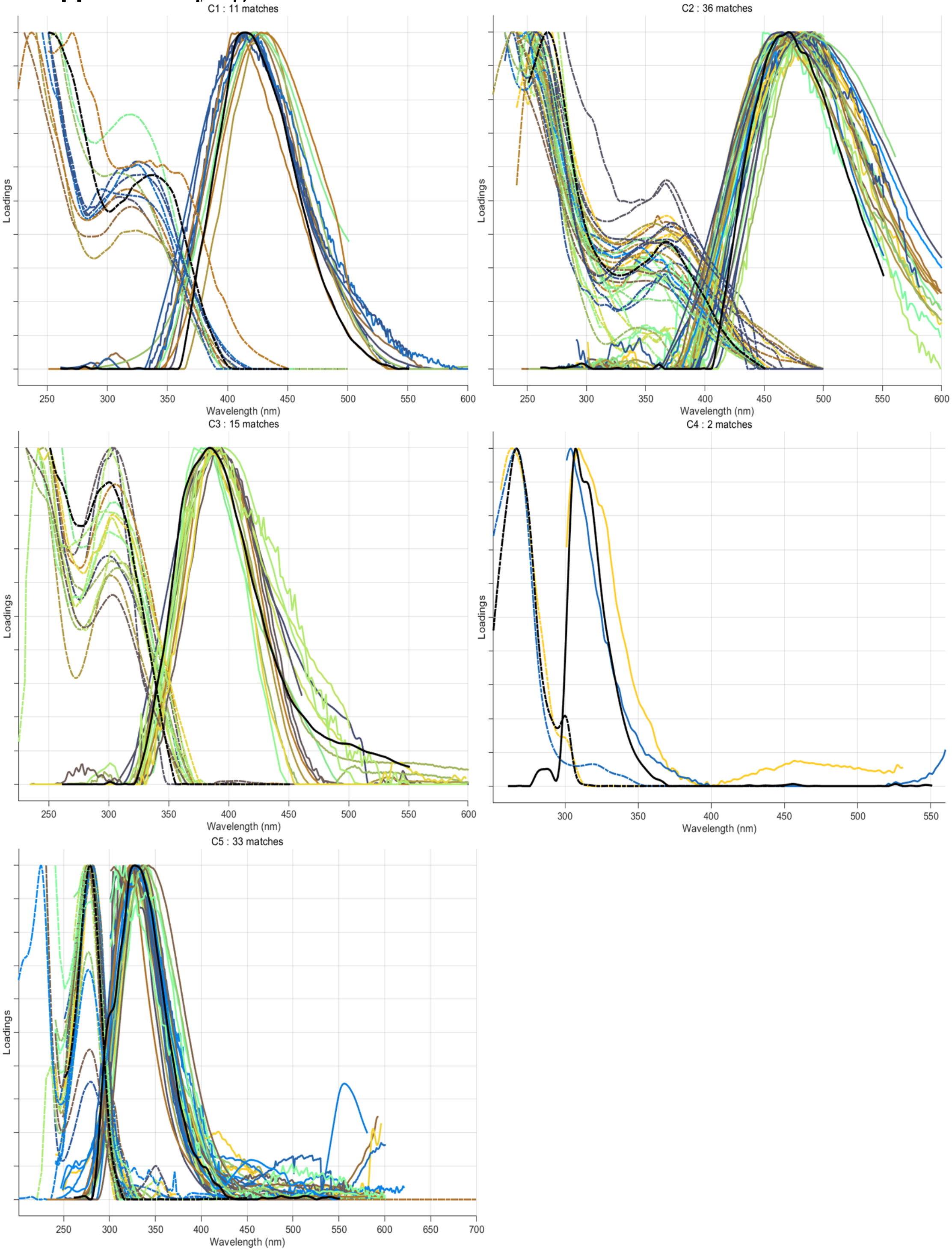

Figure S1. Comparison of a five component PARAFAC model with other models in the OpenFluor database.

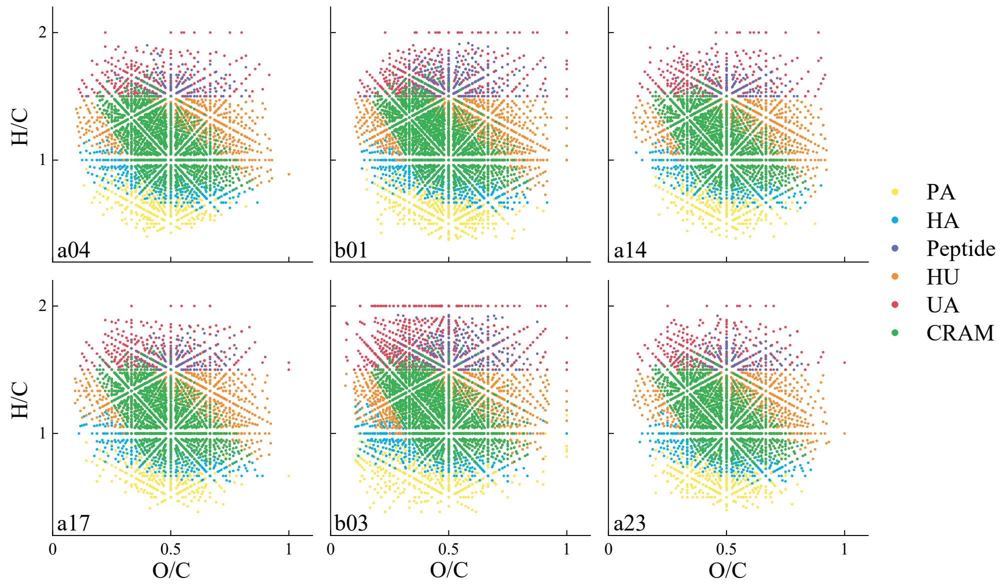

Figure S2. The upstream site a04 (a) of the Inner Mongolia section, the dividing point between the upstream and the midstream is b01 (b), the midstream site a14 (c) and a17 (d), the dividing point between the midstream and the downstream b03 (e) and the downstream site a23 (f) Van-Krevelen diagram of DOM molecular species.

2. Supplementary tables

Table S1. The coordinates of the sampling sites.

site	longitude	latitude
a01	106.75821	39.372927
a02	106.75194	39.473607
a03	106.73872	39.594979
a04	106.77411	39.641377
a05	106.75853	39.71133
a06	106.7279	39.969442
a07	106.99353	40.237236
a08	107.14128	40.379223
a09	107.39219	40.677372
a10	107.83146	40.840393
a11	108.22322	40.812539
a12	108.77514	40.609023
a13	109.45261	40.523634
a14	109.69301	40.486958
a15	109.7379	40.494965
a16	109.81894	40.512722
a17	109.93629	40.53017
a18	110.17223	40.551991
a19	110.30368	40.492117
a20	110.51171	40.390094
a21	110.65702	40.306282
a22	110.79203	40.264576
a23	110.96158	40.27816
a24	111.04583	40.295506
a25	111.20516	40.197321
a26	111.30063	40.156748
a27	111.37792	40.07396
a28	111.41519	39.997048
b01	108.73668	40.635658
b02	109.78406	40.647034
b03	109.98041	40.530991

Table S2. Element detection limit, Two-time element concentration data (Site a18, b03) and their standard deviation.

	Detection limit	Site a18			Site b03		
		Concentration-1	Concentration-2	SD	Concentration-1	Concentration-2	SD
	ug/L	ug/L	ug/L		ug/L	ug/L	
Sb	0.15	0.818	0.758	0.03	1.252	1.253	0.0005
Mo	0.06	2.532	2.937	0.2025	14.798	14.947	0.0745
As	0.12	2.472	2.516	0.022	2.17	2.275	0.0525
Zn	0.67	0.198	0.168	0.015	9.066	9.215	0.0745
Cu	0.08	1.925	1.909	0.008	1.374	1.494	0.06
Ni	0.06	0.106	0.103	0.0015	12.146	12.667	0.2605
Co	0.03	0.059	0.052	0.0035	2.008	2.052	0.022
Mn	0.12	<0.000	<0.000	--	0.69	0.785	0.0475
Cr	0.11	0.108	0.117	0.0045	0.205	0.207	0.001
V	0.08	2.088	2.168	0.04	4.588	4.569	0.0095
Al	1.15	2.891	2.841	0.025	5.705	6.084	0.1895
Se	0.41	0.606	0.618	0.006	2.939	2.598	2.939
Ga	0.02	0.023	0.016	0.0035	0.209	0.179	0.209
W	0.43	0.283	0.259	0.012	1.817	1.773	1.817
Hg	0.10	0.018	0.003	0.0075	0.020	0.013	0.020
Pb	0.09	0.045	0.007	0.019	0.053	0.036	0.053
Tl	0.02	0.005	0.002	0.0015	0.004	0.004	0.004
Zr	0.04	0.056	0.024	0.016	0.207	0.097	0.207
Ti	0.46	<0.000	0.122	--	0.205	0.087	0.205
Fe	0.82	2.710	<0.000	--	15.119	14.130	15.119
Pd	0.02	0.015	0.013	0.001	0.033	0.029	0.033

Table S3. Descriptive analysis of DOM fluorescence components and related parameters in the upper, middle, and lower reaches of the Inner Mongolia section.

Parameters	Upstream	b01	Midstream	b03	Downstream
Peak B	0.66 ± 0.10	0.56	0.66 ± 0.12	2.10	2.52 ± 0.22
Peak T	0.89 ± 0.16	0.70	0.88 ± 0.12	3.92	1.72 ± 0.18
Peak A	1.49 ± 0.13	1.44	1.59 ± 0.23	2.53	1.68 ± 0.09
Peak M	0.95 ± 0.09	0.95	1.07 ± 0.21	2.69	1.14 ± 0.06
Peak C	0.81 ± 0.08	0.82	0.86 ± 0.16	1.66	0.92 ± 0.05
Σ Peak	4.80 ± 0.44	4.47	5.06 ± 0.79	12.89	7.98 ± 0.43
C1	0.96 ± 0.09	0.91	0.97 ± 0.16	/	1.02 ± 0.06
C2	0.69 ± 0.06	0.63	0.76 ± 0.10	/	0.82 ± 0.04
C3	0.57 ± 0.08	0.61	0.70 ± 0.15	/	0.73 ± 0.04
C4	0.20 ± 0.04	0.26	0.21 ± 0.07	/	3.11 ± 0.31
C5	0.80 ± 0.17	0.58	0.76 ± 0.09	/	1.23 ± 0.18
Σ Fmax	3.21 ± 0.29	2.98	3.39 ± 0.54	/	6.93 ± 0.34
FI	2.43 ± 0.07	2.28	2.40 ± 0.04	2.91	2.35 ± 0.06
BIX	0.86 ± 0.04	0.92	0.89 ± 0.02	1.17	0.92 ± 0.03
HIX	0.76 ± 0.02	0.76	0.76 ± 0.01	0.60	0.46 ± 0.03

Table S4. The proportion of the number and relative intensity of different molecular formulas and different molecular species in the six DOM samples relative to the total number of molecules and the total intensity

Parameters	Sample					
	a04	b01	a14	a17	b03	a23
Number of formulas	4402	6719	4026	4194	6620	4166
CHO	2066	2456	1858	1951	2451	1944
% Formulas	46.93	36.55	46.15	46.52	37.02	46.66
% Intensity	74.73	66.75	72.66	73.84	53.28	73.36
CHON	1765	2576	1577	1627	2434	1645
% Formulas	40.10	38.34	39.17	38.79	36.77	39.49
% Intensity	19.63	18.44	20.81	19.78	16.71	20.36
CHOS	478	1097	483	494	1202	472
% Formulas	10.86	16.33	12.00	11.78	18.16	11.33
% Intensity	5.20	12.61	5.92	5.78	28.09	5.75
CHONS	93	590	108	122	533	105
% Formulas	2.11	8.78	2.68	2.91	8.05	2.52
% Intensity	0.45	2.20	0.61	0.60	1.93	0.53
PA	477	838	438	434	704	482
% Formulas	10.84	12.47	10.88	10.35	10.63	11.57
% Intensity	3.42	2.52	3.40	3.08	3.23	3.57
HA	710	946	593	638	849	641
% Formulas	16.13	14.08	14.73	15.21	12.82	15.39
% Intensity	9.22	6.25	8.66	8.45	5.59	8.91
HU	2578	3738	2372	2463	3473	2404
% Formulas	58.56	55.63	58.92	58.73	52.46	57.71
% Intensity	78.38	78.83	78.35	78.81	65.44	78.00
UA	464	787	457	480	1123	466
% Formulas	10.54	11.71	11.35	11.44	16.96	11.19
% Intensity	7.87	10.95	8.29	8.36	23.03	8.24
Peptide	172	404	166	179	457	172
% Formulas	3.91	6.01	4.12	4.27	6.90	4.13
% Intensity	1.11	1.40	1.30	1.30	2.57	1.28
CRAM	2320	3181	2072	2170	2980	2152
% Formulas	52.70	47.34	51.47	51.74	45.02	51.66
% Intensity	64.63	58.23	62.66	63.52	55.37	63.61

Table S5. Distribution of heavy metal concentrations in the upper, middle, and lower reaches of the Inner Mongolia section of the Yellow River Basin

Type	Parameters	**Al**	**V**	**Cr**	**Mn**	**Co**	**Ni**	**Cu**	**Zn**	**As**	**Mo**	**Sb**
	Detection limit	1.15	0.08	0.11	0.12	0.03	0.06	0.08	0.67	0.12	0.06	0.15
Upstream (n = 11)	Max	3.176	2.027	5.247	29.704	0.278	1.735	1.210	2.829	3.863	4.985	1.470
	Min	1.573	1.376	2.822	0.295	0.112	1.203	0.884	0.711	2.603	2.287	0.657
	Mean	2.053	1.743	4.643	6.374	0.164	1.429	1.019	1.444	3.299	3.073	0.981
	SD	0.420	0.238	0.695	8.852	0.045	0.140	0.099	0.625	0.402	0.761	0.250
b01	--	6.899	1.970	5.580	1.383	0.238	1.318	1.079	2.338	13.861	3.419	1.247
Midstream (n = 6)	Max	5.062	2.479	5.142	4.368	0.157	1.243	1.433	2.505	7.406	2.812	1.185
	Min	1.315	1.749	3.536	0.248	0.114	1.096	1.164	0.850	2.501	2.005	0.778
	Mean	2.427	2.096	4.005	2.401	0.136	1.161	1.344	1.542	3.554	2.441	0.903
	SD	1.297	0.287	0.524	1.327	0.014	0.047	0.088	0.579	1.731	0.273	0.141
b03	--	6.084	4.569	6.622	82.145	2.199	12.583	1.168	7.835	3.702	14.947	1.253
Downstream (n = 11)	Max	2.891	2.311	4.071	5.469	0.155	1.511	2.640	3.250	3.019	2.532	1.616
	Min	1.331	1.352	3.455	1.161	0.116	1.176	1.526	0.807	2.637	1.967	0.818
	Mean	1.909	1.737	3.679	2.542	0.135	1.281	1.812	1.942	2.803	2.132	1.001
	SD	0.462	0.295	0.195	1.392	0.013	0.111	0.387	0.724	0.126	0.175	0.255
Mainstream (n = 28)	Max	5.062	2.479	5.247	29.704	0.278	1.735	2.640	3.250	7.406	4.985	1.616
	Min	1.315	1.352	2.822	0.248	0.112	1.096	0.884	0.711	2.501	1.967	0.657
	Mean	2.078	1.819	4.109	3.930	0.146	1.309	1.414	1.669	3.154	2.549	0.972
	SD	0.736	0.298	0.660	5.793	0.033	0.150	0.423	0.657	0.908	0.644	0.219
	SV	0.354	0.164	0.161	1.474	0.223	0.114	0.299	0.394	0.288	0.253	0.225
